# Relationship between post-exercise heart rate variability and skinfold thickness

**DOI:** 10.1186/2193-1801-2-389

**Published:** 2013-08-19

**Authors:** Michael R Esco, Henry N Williford

**Affiliations:** Human Performance Laboratory, Auburn University at Montgomery, P.O. Box 244023, Montgomery, AL 36124-4023 USA

**Keywords:** Exercise, Cardiovascular, Fitness, Body composition, Autonomic nervous system

## Abstract

This investigation aimed to determine if groupings based upon sum of skinfold thickness (SF) would reflect the differences in heart rate variability (HRV) measured for up to 30-minutes following maximal exercise, and to determine the extent in variation in post-exercise HRV that could be accounted for between the following independent variables: SF, body mass index (BMI) and maximal oxygen consumption (VO2max). SF and BMI measurements were performed on fifty-four men who completed maximal exercise testing to determine VO2max. HRV was evaluated for five-minutes before (Pre), at 0-5 minutes post- (Post1) and 25-30 minutes post-exercise (Post2), and analyzed by frequency domain [high frequency (HF) power, and HF to low frequency power ratio (LF:HF)). Two groups were formed based on being above or below the sample mean value of SF. HF and LF:HF were significantly higher and lower, respectively, at Pre and Post 2 in Group 1 compared to Group 2 (p < 0.05), which remained after controlling for VO2max and BMI. Furthermore, there was a significant trend toward baseline in post-exercise HRV in Group 1 (p < 0.05) but not in Group 2 (p > 0.05). In addition, SF was the only variable to significantly relate to the post-exercise HRV parameters (p < 0.05). The findings of this investigation suggest greater SF is related to a delayed return of HRV toward baseline from maximal exercise. The association between SF and HRV is independent of VO2max and BMI.

Acute physical exertion is considered a physiological stressor with substantial effects on cardiac autonomic modulation (Hautala et al., 2001). Due to the shift in autonomic balance from parasympathetic to sympathetic dominance, it has been suggested that the heart is vulnerable to dysrhythmias and fatal events during and recovery from exercise compared to rest (Paterson, 1996; Singer et al., 1988). Furthermore, a prolonged decrease in parasympathetic tone following exercise has been associated with incomplete physical recovery (Mourot et al., 2004). Therefore, activities that accelerate parasympathetic rebound following exercise should be encouraged.

Heart rate variability (HRV) is widely used as a non-invasive measure of cardio-vascular autonomic control and is specifically predictive of cardiac dysrhythmias and sudden mortality.

(Task Force, 1996). Higher HRV has been suggested as being cardio-protective (Task Force, 1996). Indeed, spectral analysis of HRV has provided investigators a method to delineate autonomic disturbance after exercise cessation and into later stages of recovery (Hautala et al., 2001; Parekh & Lee, 2005). However, most investigations have focused on the effects of different components of exercise prescription, such as intensity (Hautala et al., 2001), modality (Heffernan et al., 2006), and duration (Parekh & Lee, 2005). To date, there is very little scientific exploration of the association of various individual physical fitness parameters on the kinetics of HRV following acute exertion.

Though resting HRV and the immediate vagal-induced heart rate recovery (HRR) following exercise appear to be related to maximal oxygen uptake (VO2max) and body composition (Aubert et al., 2003; Byrne et al., 1996; Millis et al., 2010;Molfino et al., 2009), the latter may hold an independent link to overall cardiac-autonomic control (Campos et al., 2012; Esco et al., 2011). Recently, Esco et al. (2011) showed that between selected body composition parameters and VO2max, the simplistic measure of total skinfold thickness (SF) was the strongest predictor of resting HRV and HRR when analyzed via stepwise regression. However, cardiac-autonomic activity was not measured into later stages of recovery, i.e., beyond 2-minutes (2011).

This investigation aimed to determine if groupings based upon SF would reflect the differences in HRV measured for up to 30-minutes following maximal exercise, independent of other selected markers of physical fitness, in young men. The secondary purpose of this investigation was to determine the extent in variation in post-exercise HRV that could be accounted for between the following independent variables: SF, body mass index (BMI) and VO2max. It was hypothesized that the group with the lowest SF measures would have a faster return of HRV following exercise, and that between the independent variables, SF would hold the strongest relationship with post-exercise HRV.

## Methods

### Participants

Sixty men (age = 22.6 +/- 3.2 years, height = 181.8 +/- 7.6 cm, weight = 83.0 +/- 10.7 kg) volunteered for this study. Data was collected on each subject after a 10 hour fasting period. Strenuous exercise was avoided for 24 hours prior to data collection. After receiving detailed instruction about the investigation and potential risks, each subject provided appropriate written informed consent and was informed that they could withdraw from the study at any time. Health-history questionnaires were completed. All participants that were apparently healthy, free from cardiopulmonary, metabolic, and/or orthopedic disorders, and currently not taking any prescription or over-the-counter medications were included in the study. This study was approved by the University’s Institutional Review Board for research involving human subjects.

### Body composition parameters

Height was measured with a wall-mounted stadiometer (SECA; Seca Instruments Ltd, Hamburg, Germany) and recorded to the nearest 0.5 cm. Weight was measured with a calibrated digital scale (Tanita BWB-800A, Tanita Corp, Tokyo, Japan) and recorded to the nearest 0.5 kg. Body mass index (BMI) was calculated as weight in kg divided by height in meters squared (kg.m^-2^) and rounded to the nearest 0.1 kg.m^-2^.

Skinfold measurements were obtained for each subject by the same trained technician with the use of calibrated skinfold calipers (Harpenden; Baty International, West Sussex, UnitedKingdom). For this individual, intraclass correlation coefficient within each skinfold measurement was r = 0.99. Skinfolds were measured from the following seven sites following the appropriate guidelines of the American College of Sports Medicine (2009): pectoralis major; triceps; subscapularis; mid-axillary line; abdomen; suprailliac; and thigh. All of the sites were measured in rotating order. At least two measurements were performed. If the readings were not within 2 mm of each other, a third measurement was completed. The average of the measures was calculated and rounded to the nearest 0.5 mm. The sum total of all 7 sites was recorded as SF.

### VO2max and heart rate recovery

Each subject performed a maximal graded exercise test on a Trackmaster treadmill (Full Vision, Inc., Carrollton, TX). The Bruce protocol was employed, which involved progressions in work rate (speed and grade) every three minutes until maximal oxygen consumption (VO2max) was reached. Expired gas fractions (oxygen and carbon dioxide) were collected at the mouth in a continuous manner, utilizing a mixing chamber and gas analyzers from a ParvoMedics TrueOne® 2400 metabolic cart (Sandy, UT, USA). Maximal oxygen uptake was reached if there was a plateau in oxygen consumption despite an increased work, respiratory exchange ratio of > 1.10, and a heart rate within 10 beats of age-predicted maximum (220-age). After the cessation of exercise, a 3 minute period with the treadmill workload decreased to 2.5 mph and 1.5% grade served as the cool-down. The heart rate that corresponded to VO2max was recorded as maximal heart rate (MHR). The heart rates at 1- and 2-minutes post-exercise were subtracted from MHR and recorded as HRR 1-minute (HRR1) and 2-minutes (HRR2), respectively.

### Heart rate variability

Each subject was instructed to lay supine on an athletic training table in a dimly lit laboratory for a 10-minute period before the exercise test. Immediately following the maximal exercise protocol, the subjects were allowed a 3-minute period to transition from the treadmill before comfortably assuming the supine position again on the athletic training table. The post-exercise period lasted for 30-minutes. Breathing pattern was not controlled for, as previous literature suggests that paced breathing rate masks the relationship between HRV and body fat percentage (Millis et al., 2010). The subject’s heart rate and rhythm was assessed via electrocardiogram (ECG) with surface electrodes placed across the subject’s chest in a Lead II arrangement. The electrode leads were connected to a Biopac MP100 data acquisition system (Goletta, CA, USA). All data was stored in a Dell PC. For HRV analysis, the ECG tracings were divided into three 5-minute segments as follows: the last 5 minutes of the 10-minute pre-exercise period (Pre), and at 0 to 5 minutes (Post1) post- and 25 to 30-minutes (Post2) post-exercise. For each 5-minute segment, the ECG recordings were visually inspected and any non-sinus beats were removed and replaced by the adjacent normal cycle. If three or more ectopic beats were found within any ECG segment, the subject was excluded from analysis.

Each 5-minute ECG recording was converted to a power spectrum by applying a Hanning window with a fast Fourier transformation via specialized HRV software (Nevrokard version11.0.2, Izola, Slovenia). From the power spectrum, HRV was separated into high frequency (HF) power (0.15-0.40 Hz) and low frequency (LF) power (0.04 – 0.15 Hz), which were both normalized to account for the influence of total power of the entire wave and the very low frequency band (0.0033 – 0.04 Hz). Parasympathetic-autonomic influence was considered to be represented by HF (Task Force, 1996). The LF:HF ratio was measured and recorded to indicate sympathovagal balance (Task Force, 1996). The HRV parameters were analyzed during the Pre (HFpre and LF:HFpre), Post1 (HFpost1 and LF:HFpost1), and Post 2 (HFpost2 and LF:HFpost2) time segments.

To quantify HRV kinetics in the post-exercise period (i.e., 0-5 minutes post to 25-30 minutes post), the difference in each post time point for all HRV variables were determined. The following equations were performed:

1. Post-exercise HF (HF_P-E_) = HFpost2 – HFpost1
2. Post-exercise LF:HF (LF:HF_P-E_) = LF:HFpost2 – LF:HFpost1

### Statistical analyses

Due to the previous finding of SF serving as a significant predictor of HRR independent of BMI or VO2max (Esco et al., 2011), the sample was divided into two groups as being either below (Group 1) are above (Group 2) the mean of SF for the entire sample. Means and standard deviations (SD) were determined for all the studied variables. Group differences were determined for HRR1 and HRR2 with one-way ANOVAs. To adjust for the possible independent influences of BMI and VO2max, analysis of covariance (ANCOVA) procedures were used as follow-up tests. The difference in HF and LF:HF between each measured time point (i.e., Pre, Post1, and Post2) was examined with a 2 (group) × 3 (time) repeated measures analysis of variance. Paired- and Independent-Samples T-Tests were used to further explore the time and group differences. Follow-up ANCOVAs were also used to control for potential confounders. Pearson product correlations evaluated the relationship between the selected body composition variables (i.e., BMI, and SF [as a continuous variable]), VO2max, resting HRV (i.e., HFpre, and LF:HFpre) and the post-exercise cardiac-autonomic variables (i.e., HRR1, HRR2, HF_P-E_, HF:LFC-E). Stepwise regression procedures were also used to determine which of the independent variables accounted for the greatest variation in each post-exercise cardiac-autonomic variable. Statistical significance was set at p < 0.05. Statistical analyses were performed using SPSS/PASW software (version 18.0). Data normality for all tested parameters was evaluated with a Shipiro-Wilk test. The assumption of normality was not violated for any variable.

## Results

The amount of ectopic beats exceeding the exclusion criteria were found in six subjects. Therefore, fifty-four subjects were included in data analyses. Descriptive statistics for the participants are shown in Table 1. The mean SF was 84.7 ± 30.2 mm, and thus, the two groups were divided below (Group 1, n = 27) and above (Group 2, n = 27) this value. There were statistically significant differences found between the two groups in body weight, BMI, and SF (p < 0.05). There were no statistically significant differences found in age, height, or VO2max (p > 0.05).

**Table 1:** **Descriptive statistics of the participants**

	Age (yr)	Height (cm)	Weight (kg)*	BMI (kg^.^m^-2^)*	SF (mm)*	VO_2max_(ml^.^kg^-1.^min^-1^)
Group 1 (n = 27)	22.3 ± 3.1	182.8 ± 7.3	78.6 ± 8.3	23.6 ± 1.9	62.3 ± 9.5	46.1 ± 6.6
Group 2 (n = 27)	23.0 ± 3.3	180.9 ± 7.8	87.3 ± 11.3	26.7 ± 3.1	107.1 ± 26.9	43.2 ± 7.9
Total (n = 54)	22.6 ± 3.2	181.8 ± 7.6	83.0 ± 10.7	25.1 ± 3.0	84.7 ± 30.2	44.7 ± 7.4

BMI = body mass index, SF = sum total of skinfold thicknesses, VO_2max_ = maximal oxygen consumption. *Significantly different between the two groups (p < 0.05).

Group 1 had a mean (± SD) HRR1 of 21.2 (± 6.0) beats.min-1, while Group 2 had a mean (±SD) HRR1 of 18.0 (± 6.3) beats.min-1 (p > 0.05). Group 1 had a mean (± SD) HRR2 of 46.9 (± 7.7) beats.min-1, while Group 2 had a mean (±SD) HRR2 of 38.5 (± 7.9) beats.min-1, which was significantly different (p < 0.05).

**Figure 1:**
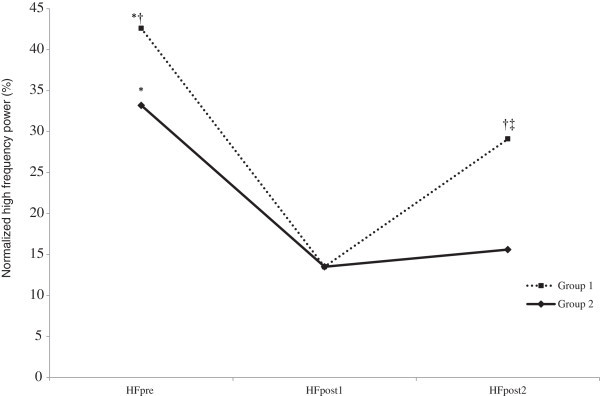
**Group × time interactions for HF.** *Significantly greater than HFpost1 and HFpost2 (p < 0.05). †Significantly greater in Group 1 compared to Group 2 (p > 0.05). ‡HFpost2 was significantly greater compared to HFpost1 in Group 1 (p > 0.05). Error bars not included for clarity.

There were significant group × time interactions for HF, which is represented in Figure 1 and values being shown in Table 2. HFpre was significantly greater in Group 1 compared to Group 2 (p < 0.05). HFpost1 and HFpost2 were significantly lower compared to the HFpre values in both groups. There were no significant group differences (p > 0.05) in HFpost1, whereas HFpost2 was significantly greater in Group 1 compared to Group 2 (p < 0.05). Furthermore, HFpost2 was significantly higher than HFpost1 in Group 1 (p < 0.05), but not in Group 2 (p > 0.05). The group x time interactions remained after controlling for VO2max and BMI (p <0.05).

**Table 2:** **Pre and post-exercise HRV (HF and LF:HF) values (means ± SD) between groups**

	HFpre (%)	HFpost1 (%)	HFpost2 (%)	LF:HFpre	LF:HFpost1	LF:HFpost2
Group 1 (n = 27)	42.6 ± 16.2*†	13.5 ± 6.7	29.1 ± 18.1†‡	1.58 ± 1.06*†	7.45 ± 5.11	3.71 ± 3.37†‡
Group 2 (n = 27)	33.2 ± 12.3*	13.5 ± 9.9	15.6 ± 11.8	2.52 ± 2.16*	7.29 ± 4.52	7.80 ± 5.28

HFpre = High frequency (HF) HRV before exercise, HFpost1 = HF HRV 0 to 5 minutes post-exercise, HFpost2 = HF HRV 25 to 30 minutes post-exercise, LF:HFpre = Low frequency to HF power HRV before exercise, LF:HFpost1 = LF:HF HRV 0 to 5 minutes post-exercise, LF:HFpost2 = LF:HF HRV 25 to 30 minutes post-exercise. *Significantly different compared to the post1 and post2 values. †Significantly different compared to Group 1 (p < 0.05). ‡Significantly different compared to post1 values (p < 0.05).

**Figure 2:**
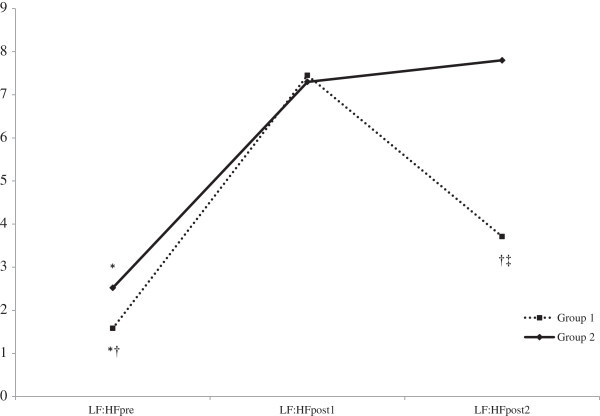
**Group × time interactions for LF:HF.** *Significantly lower in Group 1 compared to Group 2 (p > 0.05). †Significantly greater in Group 1 compared to Group 2 (p < 0.05). ‡LF:HFpost2 was significantly lower compared to LF:HFpost1 in Group 1 (p > 0.05). Error bars not included for clarity.

There were significant group × time interactions for LF:HF, which is represented in Figure 2 and values being shown in Table 2. LF:HFpre was significantly lower in Group 1 compared to Group 2 (p < 0.05). LF:HF was significantly higher at Post1 and Post2 compared to Pre values in both groups (p < 0.05). There was no significant group difference (p > 0.05) in LF:HFpost1, whereas LF:HFpost2 was significantly lower in Group 1 compared to Group 2 (p < 0.05). Furthermore, LF:HFpost2 was significantly lower compared to LF:HFpost1 in Group1 (p < 0.05), but not in Group 2 (p > 0.05). The group × time interactions remained after controlling for VO2max and BMI (p <0.05).

Table 3 represents the zero-order correlation coefficients between the independent and dependent variables. There were significant correlations found between SF and HRR2 (r = - 0.35, p < 0.05), HFpre (r = -0.28, p < 0.05), HF_P-E_ (r = -0.35, p < 0.05), and LF:HF_P-E_ (r = 0.35, p < 0.05). Neither VO2max nor BMI significantly correlated with any HRR or HRV variable (p > 0.05). In addition, there were no significant correlations found between the independent variables and HRR1 or LF:HFpre (p > 0.05). The results of the stepwise regression procedures showed that SF accounted for the greatest variation in HRR2 (R2 = 0.12, p < 0.05), HF_P-E_ (R2 = 0.12, p < 0.05), and LF:HF_P-E_ (R2 = 0.12, p < 0.05). Neither VO2max nor BMI added statistical significance to the regression models, above and beyond that of SF.

**Table 3:** **Zero-order correlation coefficients (r) showing the relationship between the variables**

	VO_2max_	BMI	SF
HRR1 (beats^.^min^-1^)	−0.02	−0.02	−0.16
HRR2 (beats^.^min^-1^)	0.15	−0.15	−0.35*
HF_P-E_	−0.06	−0.16	−0.35*
LF:HF_P-E_	−0.23	0.12	0.35*

VO_2max_ = maximal oxygen consumption; BMI = body mass index; SF = sum of skinfold thickness; HRR1 = heart rate recovery 1-minute post-exercise; HRR2 = heart rate recovery 2-minutes post-exercise; HF_P-E_ = high-frequency power (HF) heart rate variability (HRV) 25 to 30 minutes post-exercise minus HF HRV 0 to 5 minutes post-exercise (i.e., HFpost2 – HFpost 1); LF:HF_P-E_ = HF to low frequency power (LF) HRV ratio 25 to 30 minutes post-exercise minus HF to LF ratio 0 to 5 minutes post-exercise. *Significantly related, p < 0.05.

## Discussion

The results of this study suggest that SF, but not VO2max or BMI, is significantly related to cardiac-autonomic regulation at rest and post-exercise. The finding of a relationship between SF and resting HRV and HRR is in agreement with previous research (Esco et al., 2011). In addition, Campos et al. (2012) showed that HRR was linked to body fat percentage, but not VO2max, which also concurs with our findings since the SF technique is commonly used to predict body fat percentage in field settings (ACSM, 2009). The most important finding from the current study extends previous research, showing a significant and independent association between SF and HRV that was measured up to 30-minutes following exercise.

The study’s sample was cross-sectionally divided into two groups based upon being either below (i.e., Group 1) or above (Group 2) the mean SF. Group 1 had a significant trend toward baseline in all of the post-exercise HRV markers, as HFpost2 and LF:HFpost2 were significantly different from HFpost1, and LF:HFpost1, respectively. In contrast, Group 2 did not have a significant trend toward baseline in the frequency domain parameters, as there were no significant differences in the Post1 and Post2 values. The group differences in the post-exercise HRV values remained after controlling for BMI and VO2max, showing the independent effect of the SF groups. These findings suggest a faster post-exercise return of HRV in the leaner group of men. Thus, the first hypothesis of the study was supported.

In addition, the relationship between SF (as a continuous variable), BMI, VO2max, and the cardiac-autonomic measures was also determined. It was found that SF was the best predictor of resting HRV and HRR which. Most importantly, however, SF was the only variable to relate to the return toward baseline in HRV from immediate to 30-minutes post-exercise (i.e., Post2 minus Post1: HF_P-E_ and LF:HF_P-E_). In other words, SF significantly and independently explained 12% of the variation in HF_P-E_ and 11% of the variation in LF:HF_P-E_. Neither BMI nor VO2max were related to the post-exercise HRV values. Thus, the findings support the second hypothesis that SF is independently associated with post-exercise HRV.

Physical exertion acutely increases cardiac output and results in a redistribution of blood flow to meet the metabolic demands of active skeletal muscle. After exercise, there is a prompt decrease in cardiac output as physiological activity returns towards resting conditions. Specialized afferent receptors, such as metaboreceptors and baroreceptors, are highly involved with the changes in cardiovascular-autonomic modulation during and after exercise. However, increased intramuscular fat reduces skeletal muscle uptake of glucose and attenuates acidosis during exercise (Sherman et al., 1988), and therefore, could lower the activation of the metaboreceptors (Dipla et al., 2012). Furthermore, reduced baroreflex sensitivity following brisk walking has been reported in obese compared to lean women (Figueroa et al., 2007a). Consequently, it appears that the afferent feedback mechanisms that normally result in an appropriate autonomic adjustment of the cardiovascular system during and following exercise is impaired with a higher amount of body fat (Dipla et al., 2012), which help to explain the results of the current study.

The finding of no relationship between BMI and any autonomic parameter was expected. Though BMI is moderately related to chronic disease risk factors, body fat percentage appears to be overall a better predictor of such conditions (Zeng et al., 2012). The primary reason for this is due to the disadvantage of BMI failing to distinguish between lean and fat tissues (Nevill et al., 2006; Zeng et al., 2012). Furthermore, previous research has shown significant associations between resting HRV and markers of body fat percentage, but not BMI (Esco et al., 2011; Millis et al., 2010), which support the current findings.

The finding of no association between VO2max and post-exercise HRV is perplexing, since an improvement in resting vagal tone usually occurs with endurance training (Shin et al., 1997). However, this is in agreement with Cornelissen et al., (2010) who showed no change in post-exercise HRV following 10-weeks of exercise, though VO2max increased. Yamamoto et al. (2001) showed an improvement in post-exercise HRV after only 1-week of endurance training, though no changes were found between weeks 2 and 6. Therefore, endurance training may result in a minimal enhancement of post-exercise HRV. Other improvements in physical fitness, compared to just aerobic conditioning, could also play an important and additive role.

Changes in body composition have been shown to enhance autonomic tone at rest and in response to stress (Ashida et al., 2007; Blumenthal et al., 2010; de Jonge et al., 2010; Figueroa et al., 2007b; Grassi et al., 1998; Tonacio et al., 2006; Trombetta et al., 2003). Diet induced weight loss improved baroreceptor sensitivity and metaboreflex in obese subjects (Grassi et al., 1998; Trombetta et al., 2003). In addition, Tonacio et al. (2006) showed a significantly greater improvement in forearm vascular conductance following mental stress in a group of obese women who lost weight from a diet-plus-exercise intervention compared to a group who lost a similar amount of weight from a diet-only intervention. Other investigators have shown an improvement in autonomic regulation with weight loss from caloric reduction (Ashida et al., 2007), and an even greater effect with the addition of exercise (Blumenthal et al., 2010; de Jonge et al., 2010). Furthermore, 16 weeks of endurance exercise training improved post-exercise HRV, baroreflex sensitivity, and peak maximal oxygen consumption in obese women, without a concomitant significant change in body composition (Figueroa et al., 2007a). Therefore, it is reasonable to consider that lifestyle interventions designed to influence both aerobic fitness *and* body composition could result in the greatest enhancement of autonomic control of heart rate. Research determining the effects of chronic exercise training with and without a reduction in body fat on resting and post-exercise cardiac-autonomic modulation is needed.

Unfortunately, the cross-sectional and correlational design of the study limits the ability to determine cause-and-effect. Furthermore, the current study only included young-adult men. Therefore, it is difficult to extrapolate the findings to consider women and older subjects. Cardiac-autonomic control has been shown to be different between sexes and influenced by aging (Vandeput et al., 2012). In addition, the sample was grouped based on whether they were above or below the mean skinfold. The main emphasis within public health is place in the extremes of the distribution; i.e., with significantly underweight or obese. However, the novel finding of the current study provides an important first step for future research in subjects with clinical weight-related issues.

## Conclusions

The findings of this investigation suggest greater SF is related to a delayed return of HRV toward baseline from maximal exercise. Therefore, healthy body fat percentage could be cardio protective during the post-exercise period, when the heart is at an immediate risk of an unfavorable event. Practitioners may need to consider targeting a reduction in body fat percentage with lifestyle interventions when attempting to enhance cardiac-autonomic control.
